# Oral Microbial Diversity Formed and Maintained through Decomposition Product Feedback Regulation and Delayed Responses

**DOI:** 10.1155/2021/5590110

**Published:** 2021-02-20

**Authors:** Chen Dong, Dandan Li, Zengfeng Wang, Zhengde Bao

**Affiliations:** ^1^Laboratory of Sport Nutrition and Intelligent Cooking, School of Sport Social Science, Shandong Sport University, Jinan 250102, China; ^2^Office of Educational Administration, Qingdao University of Science & Technology, Qingdao 266061, China; ^3^Jincheng College, Sichuan University, Chengdu 611731, China

## Abstract

Oral microbial diversity plays an important role on oral health maintenance. However, there are only few kinds of substrates available for the microbial flora in oral cavity, and it still remains unclear why oral microbial diversity can be formed and sustained without obvious competitive exclusion. Based on experimental phenomena and data, a new hypothesis was proposed, namely, the decomposition product negative feedback regulation on microbial population size and microbial delay responses including reproductive, reaction, interspecific competition, and substrate decomposition delay responses induced by oral immunity. According to hypothesis and its cellular automata (CA) model, the CA simulation results sufficiently proved that the decomposition product negative feedback regulation and four microbial delay responses could significantly alleviate the interspecific competitions and inhibit the emergence of dominant species, causing the formation and sustenance of oral microbial diversity. This study could also offer effective guidance of prevention and treatment of oral cavity diseases.

## 1. Introduction

As is known, oral microorganisms are one of the five major microbial florae of the human body, oral microbial diversity is an important indicator to evaluate the oral health status of people, and simultaneously, it also closely related to systemic diseases occurrence, such as digestive system [1], movement system [2], nervous system [3, 4], and circulatory system [5], playing a significant role in our health care [6].

Oral microorganisms come mainly from outside environment through the air, water, and food and colonize different places of oral cavity, such as saliva, gingiva, and oral walls. Compared with substrates in natural environments, however, the types and amounts of substrates in oral cavity can provide for microbial growth is extremely limited. According to the competitive exclusion principle in ecology, microbial species would have to compete fiercely over few varieties of substrates under this circumstance, and only a few microbial species can coexist via substrate niche differentiation, which is unfavorable for microbial diversity [7, 8]. Why oral microbial diversity can be formed and maintained in a way apparently violating competitive exclusion principle remains mysterious so far [9].

In the research, 50 mixed-gender athletes with healthy oral cavity were randomly selected as testers, and the abundance, intracellular triglycerides, and specific growth rates of the five common genera, *Streptococcus*, *Prevotella*, *Haemophilus*, *Rothia*, and *Veillonella* were analyzed and obtained through periodical sampling from their oral floras. The results show that intracellular triglycerides of all genera were significantly higher than their homogenous genera in natural environment, and it has been reported that the microbial species could only utilize the intracellular energy substances to grow independent of ambient substrates (Wilkinson, 1963). Nevertheless, there were quite different specific growth rates between genera, and the abundances consistently stayed in unstable states with a tendency of asynchronously convergent fluctuations and low Simpson *α* diversity index. Through these experimental phenomena and data, two fundamental oral microbial delay responses including reproductive delay and reaction delay were identified based on delay logistic equation and digital simulation, which are inevitably accompanied by other two delay responses, i.e., interspecific competition delay and LMOM decomposition delay according microbial ecology.

As we know that microbes need to break down large molecular organic matters (LMOMs) into small molecular organic matters (SMOMs) that can be directly absorbed and assimilated by cells, such as starches and celluloses were broken down into monosaccharides and oligosaccharides, proteins into oligopeptides and amino acids, fat into glycerol and fatty acids. The processes of decomposing LMOMs into SMOMs need to greatly consume metabolic energy and often take place in the extracellular environments, whereas SMOMs are absorbed and assimilated inside the cells. Hence, SMOMs could be considered as the public goods that could directly be utilized by all microbial species [10, 11].

Therefore, in the article, a new dynamic mechanism of oral microbial diversity formation and maintenance was put forward as follows:

According to adaptabilities to the oral environment, the oral microbial species could be divided to two types, *collaborators* and *scammers*. As colonized in oral cavity, *collaborators* could well adapt to oral environment and get an enhanced LMOM decomposition ability, resulting in accelerated growth of *collaborator* populations, and simultaneously more SMOMs can also be produced. In contrast, *scammers* cannot acclimate to the oral environment without enhancement of LMOM decomposition capacities. As the *collaborators* and *scammers* are combined into an oral microbial flora, the *scammers* might be more prone to utilizing these ready-made SMOMs produced by *collaborators*. Since the *collaborators* would have to entail the high cost of LMOM decomposition, the *scammers* would pay nothing to obtain SMOMs; hence, *scammers* could easily win in the interspecific competition and competitively exclude *collaborators* gradually [12]. Once the population of *collaborators* drops drastically, however, SMOM decrease would ensue to undoubtedly hinder the further growth of the *scammer* population due to starvation. Hence, an SMOM-based negative feedback regulation on microbial population size might exist in oral microbial community. If the *collaborators* went extinct and *scammers* would be eradicated inexorably, the oral microbial diversity could not be formed and sustained in oral cavity at all. Owing to existence of abovementioned four delay responses, however, both *collaborators* and *scammers* would not go extinct at all. The SMOM-based negative feedback regulation and delay responses would drive the oral microbial flora succession with asynchronously convergent fluctuations of populations. Referring to the classic *Lotka–Volterra* equations, the interspecific competition intensity mainly depends on the product of population size, and the asynchronously convergent fluctuations of microbial populations could significantly alleviate the interspecific competitions and inhibit the emergence of dominant species, forming and maintaining the oral microbial diversity by a strategy of species-for-quantity exchange.

Based on the assumptions and experimental data, a highly valid cellular automata (CA) model was established to describe oral microbial community succession, and its local rules could sufficiently represent the SMOM-based negative feedback regulation on microbial population size and the four delay responses. The digital simulation results confirmed the hypothesis proposed undoubtedly from the view of time and space dimensions simultaneously, and this study can lay the theoretical foundation to understand the mechanism of the forming and maintenance of microbial diversity in oral cavity, offering effective guidance of prevention and treatment of oral cavity diseases.

## 2. Materials and Methods

### 2.1. Source of the Samples

The 50 students with mixed-gender and aged 18 to 25 were randomly selected from undergraduates and postgraduates in Shandong Sport University.

All participants should meet the following inclusion criteria during the experiment:

  Without periodontitis, oral mucosal diseases, dental caries, and other oral diseases
  Without systemic diseases and behavioral disorders
  Physical and psychological indicators were basically normal
  Nonsmoking and alcohol-free
  No drug dependence or history of drug addiction
  Not using antibiotics in the past 3 months
  No more than 2 missing teeth with the exception of extracted third molars
  Mean clinical attachment level (CAL) ≤0.5 mm, no interproximal sites with CAL ≥ 3 mm [13]

Besides, all participants were asked to rinse their mouths with sterile saline (0.9%) for 1 to 2 min to remove the debris. No eating, drinking, smoking, or chewing gum during this period. Each participant kept saliva in the mouth for at least 1 min, chewed the swab from a saliva sampling tube (Salivette®, SARSTEDT) and placed the swab back into the tube. The entire tubes were then centrifuged at 10,000 rpm for 5 min. After discarding the supernatant, the collected cells were used as samples for measurement and analysis of the relative abundances, intracellular triglycerides, and specific growth rates of the five most common genera, *Streptococcus*, *Prevotella*, *Haemophilus*, *Rothia*, and *Veillonella*.

### 2.2. Determination of Intracellular Lipids of Oral Microorganisms

To compare of the intracellular storage substance content of oral microorganisms in the external and oral environment, the cells in above samples were resuspended in 1 ml of 0.1 mol L^−1^ phosphate buffer. The test solution was again centrifuged with the above parameters after ultrasonic fragmentation. The intracellular lipid content was determined using a triglyceride assay kit and analyzed by a UV spectrophotometer at 420 nm.

### 2.3. Determination of Specific Microbial Growth Rate

The cells in above samples were also resuspended in 2 mL of sterile saline (0.9%), for high-throughput sequencing to obtain relative changes in the numbers of different populations. The main steps are as follows: (1) DNA extraction, the genome DNA was extracted by column genomic DNA extraction kit, and the integrity of the extracted genomic DNA was tested using 1% agarose gel electrophoresis. (2) 16S rDNA amplification, then, the primers were obtained according to the conserved region design, and sequencing barcodes were added at the end of the primers for PCR amplification. The PCR amplification primers used were 515F: GTGCCAGCMGCCGCGG and 907R: CCGTCAATTCMTTTRAGTT. PCR reaction conditions were *a*: 2 min, 95°C; 1 time cycle; *b*: 30 s, 95°C; 30 s, 55°C; 45 s, 72°C; 28 cycles; and *c*: 10 min, 72°C, 4°C until termination. (3) After recovery and purification of amplified products, DNA libraries were constructed and their quality was evaluated, and sequencing of the qualified libraries was performed on the platform Illumina HiSeq 2500. The original image data files were converted into sequenced reads by base calling analysis, and the results were stored in FASTQ file format, which contained the sequence information of reads and the corresponding sequencing quality information [14].

Based on the experimental data of exponential growth stage, the growth rate of microbial population could be defined as follows:

(1) $$\begin{array}{c} \frac{dx}{dt}=\mu x, \end{array}$$

where *x* is the microbial population at *t*, *μ* is the specific growth rate, and the *μ* could be calculated by *μ*=Δ*x*/*x*Δ*t* from the corresponding discrete form of equation (1).

### 2.4. Delay Response Identification

The delay logistic equation equation (2) was used to identify the fundamental delay responses (Ellermeyer et al, 2003):

(2) $$\begin{array}{c} \frac{dx}{dt}=\mu x\left(t-\tau_{1}\right)\left[1-\frac{x\left(t-\tau_{2}\right)}{K}\right], \end{array}$$

where *x* and *μ* have the same meaning as they are in equation (1) and *τ*_1_ and *τ*_2_ are microbial reproductive delay and reaction delay, respectively. In the research, dynamic response optimization was used to identify these two fundamental delay responses, based on experimental data and digital simulation on the platform of Matlab/Simulink.

### 2.5. CA Modeling and Simulation

Because oral cavity is peculiar ecosystem, the relationships and interactions between microbial species and their biotic/abiotic environment are extremely complicated with strong nonlinearity and uncertainty, and it is difficult to carry out prototype experiments or analytical and numerical methods for investigation and elucidation of the dynamic mechanisms to drive oral microbial flora succession. The CA modeling and simulation has been extensively applied for theoretical investigation of complex systems, such as medicine, biology, and sociology. Based on local rules, CA can simulate extremely complicated structure and dynamic behaviors to predict the unexpected holistic emerging characteristics which cannot be realized by traditional ordinary and partial differential equation modeling at all [15]. Therefore, a highly valid CA models were developed and digital simulations were conducted to obtain the general pattern of spatiotemporal succession in combination with system cluster analysis, since its local update rules could fully embody preceding two decisive succession mechanisms, i.e., the decomposition product negative feedback regulation on microbial population size and four microbial delay responses induced by oral immunity.

## 3. Results and Discussion

### 3.1. Microbial Response Characteristics to Oral Environment

#### 3.1.1. Relative Abundances and Diversity Dynamic Characteristics

**Figure 1:**
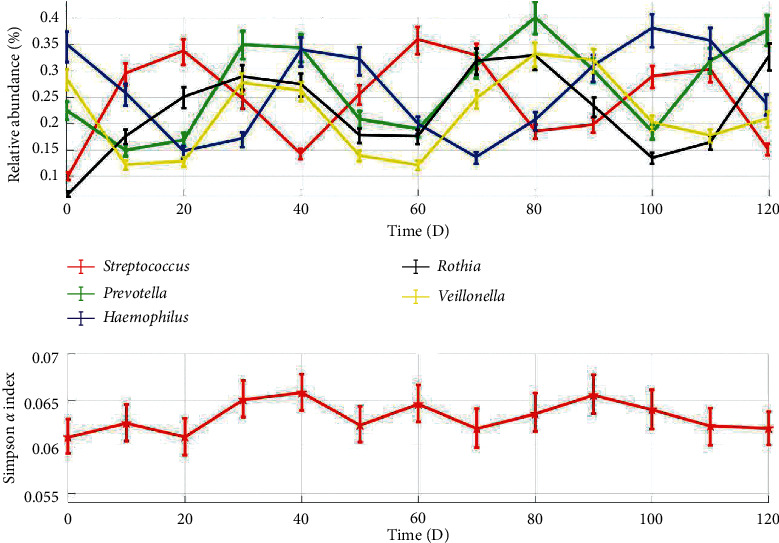
Dynamic characteristics of microbial populations and Simpson *α* diversity in oral cavity.

The relative abundances of *Streptococcus*, *Prevotella*, *Haemophilus*, *Rothia*, and *Veillonella* in oral cavity and corresponding Simpson *α* diversity were obtained via periodical samplings and analyses (Figure 1).

As illustrated in Figure 1, these time-series data showed apparently the oral microbial flora consistently stayed in unstable states with asynchronous convergent fluctuations of microbial populations and high evenness [16].

#### 3.1.2. Intracellular Triglycerides and Specific Growth Rate

**Figure 2:**
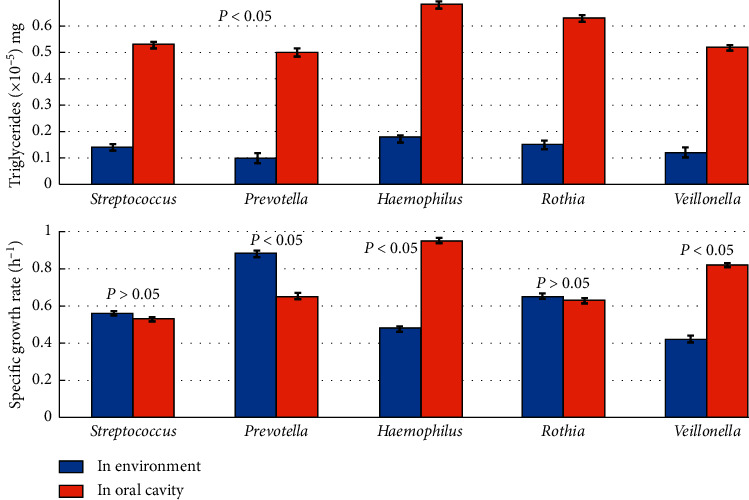
Variation of intracellular triglyceride content and specific growth rate of microbial populations in natural environment and oral cavity.

As illustrated in Figure 2, the intracellular triglycerides of all oral microbial genera were significantly higher than their homogenous strains existing in natural environment. In terms of specific growth rate, however, compared with their counterparts in natural environment, *Haemophilus* and *Veillonella* are significantly higher, *Prevotella* is significantly lower, and *Streptococcus* and *Rothia* have no significant difference.

#### 3.1.3. Identification of Microbial Delay Responses

**Figure 3:**
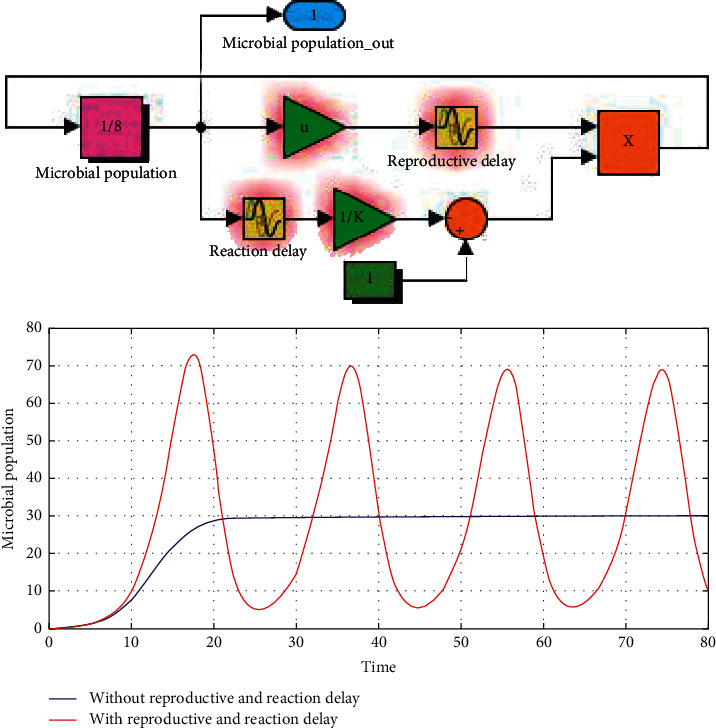
Microbial population dynamic response characteristics without and with delay effects.

Generally, along with the reproductive delay (*τ*_1_) and the reaction delay (*τ*_2_) increase in delay logistic equation (Equation (1)), the population dynamic characteristics vary from asymptotical stabilization to convergent fluctuation (Figure 3).

Based on experimental data (Figure 1), delay logistic equation (Equation (1)), and digital simulations (Figure 3), the *τ*_1_ and *τ*_2_ of *Streptococcus*, *Prevotella*, *Haemophilus*, *Rothia*, and *Veillonella* were precisely identified through dynamic response optimization (Table 1).

#### 3.1.4. Hypothesis of Formation and Maintenance of Microbial Diversity in Oral Cavity

Based on oral microbiology, microbial ecology, phenomena observed, and experimental data (Figures 1–3), a new assumption of oral microbial diversity formation and maintenance was proposed as follows.

Although microbial species have capabilities to decompose LMOMs into SMOMs for survival in natural environment, as colonized in oral cavity, some microbial species (called *collaborators*), such as *Haemophilus* and *Veillonella*, could well adapt to oral environment and get an enhanced LMOM decomposition ability resulting in accelerated growth and simultaneously more SMOMs could be produced. In contrast, other species (called *scammers*), such as *Streptococcus*, *Rothia*, and *Prevotella*, do not have such adaptively physiological and behavioral response characteristics to oral environment, which reflected in the specific growth rate of *Streptococcus* and *Rothia* had no change, and *Prevotella* growth was significantly inhibited, and it also indicated that their LMOM decomposing capacity could not be strengthen in oral environment.

Since the *collaborators* would have to entail the high cost of LMOM decomposition, they would benefit more from cooperation than competition, and interspecific cooperation begins to dominate. However, the *scammers* would pay nothing to obtain SMOMs, so *scammers* could easily win in the interspecific competition to competitively exclude *collaborators* [12]. Once the *collaborator* population drops drastically, the accompanying decrease of SMOMs appears inexorably and would undoubtedly hamper the further growth of the *scammer* population due to starvation. If the *collaborators* went extinct, *scammers* would be extirpated, and oral microbial diversity could not be formed and sustained at all.

From Figure 2, we might speculate that, as SMOM is plentiful, both *collaborators* and *scammers* could assimilate SMOMs which could convert into intracellular energy substances such as triglycerides, in order to overcome adversity in the future. At this time, microbial species might stay in time-lag state of reproduction and reaction [17, 18], and microbial cells did not divide until environmental factors such as SMOM amount and interspecific competition strengths were suitable for them in the oral environment.

Based on the above analysis, two crucial fundamental succession mechanisms might exist in oral microbial flora as follows:

1. The SMOM-based negative feedback regulation on microbial population size: as the population of the *collaborators* increases, which is followed by accumulation of SMOMs, the *scammer* population will also grow and inevitably exert competitive exclusion against *collaborators*, causing the *collaborator* population drop drastically and the accompanying decrease of SMOMs appears, which undoubtedly hinders the further growth of the *scammer* population and reduce the intraspecific competition between *scammers* and the strength of competitive exclusion to *collaborators.* Since the *scammer* population declines, the *collaborator* population rebounds. At this time, the microbial community succession seems to return to the original point and completes a cycle. Hence, SMOMs might play a role on a negative feedback regulation of microbial population to cause *collaborator* and *scammer* population fluctuation.
2. The delay responses of microbial species: the reproductive and reaction delay responses were produced to acclimate for oral environment mainly by intracellular energy substance storage. Theoretically, these two fundamental delays must be accompanied by interspecific competition delay and LMOM decomposition delay. These four delay effects would further exacerbate population fluctuations [18, 19].

Referring to the classic *Lotka–Volterra* equations, the interspecific competition intensity only depends on the product of their population size in the case of the competition coefficient unchanged; hence, these two decisive dynamic mechanisms could give rise to asynchronously convergent fluctuations of microbial populations, which can significantly alleviate the interspecific competitions and inhibit the emergence of dominant species, causing formation and maintenance of the microbial diversity with higher richness and evenness by a strategy of species-for-quantity exchange.

Based on preceding hypotheses, a valid CA model describing oral microbial flora spatiotemporal succession was developed and a great number of digital simulations were conducted to confirm proposed hypotheses.

### 3.2. CA Modeling and Simulation of Oral Microbial Flora Spatiotemporal Succession

#### 3.2.1. Cells

A cell represents a microbial individual of a certain species with 4 states as follows:

(3) $$\begin{array}{c} S=\left(\text{Pos},\text{ Spe},\text{ isLag},\text{ Lat},\text{ Clr}\right), \end{array}$$

where

1. Pos (*i*, *j*) denotes whether or not a position (*i*, *j*) was occupied by a microbial individual, and **1** and **0** represent “occupied” and “unoccupied”, respectively.
2. Spe (i, j) denotes the type of a microbial individual at position (i, j), and 1 and 0 represent “*collaborators*” and “*scammers*”, respectively.
3. isLag (i, j) denotes whether or not a microbial individual at position (i, j) stays in time-lag state, and 1 and 0 represents “yes” and “no”, respectively.
4. Lat (i, j) records the lag time of a microbial individual at position (i, j).
5. Clr (i, j) denotes the color of a microbial individual at the position (i, j), specified by an RGB value.

#### 3.2.2. Cellular Space and Boundary Conditions

**Figure 4:**
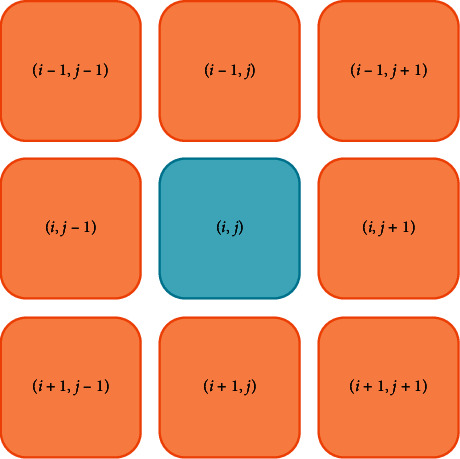
CA neighbor type.

1. Lattice: 2D domain with *10*^*3*^ × *10*^*3*^ uniform square meshes.
2. Neighbor type: Moore-type was applied for CA modeling and simulation (Figure 4), each cell has 8 neighboring cells.
3. Boundary conditions: periodic boundary.

In order to obtain a general pattern of microbial community spatiotemporal succession in oral cavity, periodic boundary was used for CA simulation, indicating cellular space was connected up and down and left and right to form a torus structure, which could be considered as an infinite cellular space extensively applied for theoretical investigation.

#### 3.2.3. Update Rules

The key part of the CA modeling is update rules sufficiently embodying the two fundamental dynamic mechanisms driving oral microbial community succession as follows:

1. *Delay Response Rules*. For a microbial individual at position (*I*, *j*) and time *t*, its delay response is dependent on the total sum, MLL (*I*, *j*), of *collaborator* and *scammer* individuals in the nearest neighborhood:

(4) $$\begin{array}{c} \text{MLL }\left(i,j\right)=\text{Spe }\left(i-1,j-1\right)+\text{Spe }\left(i,j-1\right)+\text{Spe }\left(i+1,j-1\right)+\text{Spe }\left(i-1,j\right)+\text{Spe }\left(i+1,j\right)+\text{Spe }\left(i-1,j+1\right)+\text{Spe }\left(i,j+1\right)+\text{Spe }\left(i+1,j+1\right). \end{array}$$

  For a *collaborator* individual at position (*i*,  *j*), if its neighboring *scammer* individuals meet 6 ≤ MLL (*i*, *j*) ≤ 8, 3 ≤ MLL (*i*, *j*) ≤ 5, 0 ≤ MLL (*i*, *j*) ≤ 2, then it would enter lag phase with probability *α*_*1*_, *α*_*2*_, *α*_*3*_, respectively, satisfying *α*_*1*_ > *α*_*2*_ > *α*_*3*_.
  For a *scammer* individual at position (*i*, *j*), if its neighboring *collaborator* individuals meet 6 ≤ MLL (*i*, *j*) ≤ 8, 3 ≤ MLL (*i*, *j*) ≤ 5, 0 ≤ MLL (*i*, *j*) ≤ 2, then it would enter lag phase with probability *β*_*1*_, *β*_*2*_, *β*_*3*_, respectively, satisfying *β*_*1*_ < *β*_*2*_ < *β*_*3*_.
  It is also worth pointing out that a microbial individual staying in lag phase is similar as the dead one, except for the former needs to occupy a position (Pos (*i*, *j*) = 1), and the latter will release space (Pos (*i*, *j*) = 0). Once *collaborator* and *scammer* individuals stay in lag phase, they would not decompose LMOMs, absorb and assimilate SMOMs, compete or cooperate with other neighboring individuals, and divide to create offspring to occupy other positions. The microbial individual would recover from the lag phase; however, the microbial individual would come to death with a probability *p* as long as the lag time exceeds maximum time-lag phase, Lat (*i*, *j*) × *n*, where *n* is an integer, for depletion of intracellular energy substance storage.
(2) *Rule of Birth and Death.* Although the life and death of microbial individuals is mainly dependent on the interspecific competition intensity of the nearest neighboring individuals, the *collaborator* and *scammer* individuals would die off naturally with probability *d* (*d* *<* *p*) at each time step.
(3) *Rule of Move.* This rule expresses microbial cell proliferation with the moving radius of 3. If a position, Pos (*i*, *j*), is vacant, its neighboring 48 individuals of three layers centered on Pos (*i*, *j*) could move to this position with the same probability *m.*

### 3.3. CA Simulation of Oral Microbial Flora Spatiotemporal Succession

#### 3.3.1. Spatial Pattern of Oral Microbial Flora Succession

The *N* kinds of microbial species including *n*_*1*_ kinds of the *collaborators* and *N*-*n*_*1*_ kinds of the *scammers* are computer-generated to completely random seeding on the grids of cellular space. Since a microbial species could be considered as a characteristic parameter vector with set intervals (Table 2), hence *N* kinds of microbial species could be obtained by uniformly and independently random selection from these parameter intervals through Monte Carlo experiments and set to the CA model for simulation. For example, a *collaborator* species could be defined as a parameter vector [*α*_*1*_, *α*_*2*_, *α*_*3*_, *β*_*1*_, *β*_*2*_, *β*_*3*_, *m*, *p*, *d*, *Lat*, *n*] whose magnitude was mapped into interval of [0,1] and then assigned to Clr (*i*, *j*), causing color change of a grid in lattice occurred.

**Figure 5:**
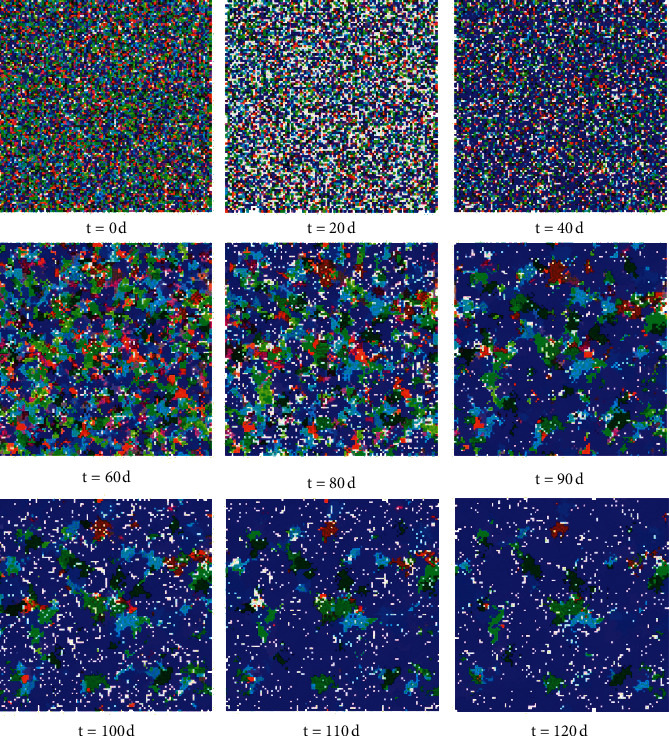
Pattern of microbial community spatiotemporal succession process in oral cavity.

Driven by SMOM feedback regulation of microbial population and microbial delay responses, the spatial pattern of oral microbial flora succession is illustrated in Figure 5. Because the relatedness of the microbial individuals is embodied in color similarity between them, they showed apparently a specific spatial pattern of aggregated distributions.

**Figure 6:**
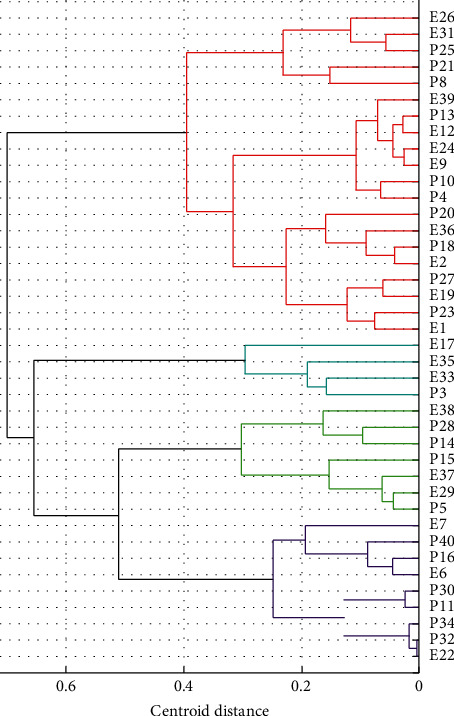
Cluster analysis of climax microbial community resulting from CA simulation in oral cavity.

Therefore, system cluster analysis was conducted to investigate the similarity of microbial individuals in these patches, and the *Minkowski* method and *Centroid* method were used to measure the distance of two microbial characteristic vectors and generate a hierarchical cluster tree (Figure 6), respectively, since these two methods corresponded to maximum *cophenetic* correlation coefficient (0.97). Hence, it was concluded that the microbial individuals were randomly scattered on the grids in the beginning stage of succession, while microbial individuals with closer affinities began to gradually aggregate to form patches, along with succession process.

The CA simulation results were highly similar to the phenomena observed in the experiments [20], i.e., microbial species with a close relationship locally tended to aggregate in patches, which could effectively ease the interspecific competitions to be propitious to form and maintain the microbial diversity.

#### 3.3.2. Time-Domain Response Characteristics of Oral Microbial Flora Succession

In order to confirm the role of microbial delay responses on formation and maintenance of oral microbial diversity, parameters closely relevant to time-lags, such as *α*_*1*_, *α*_*2*_, *α*_*3*_, *β*_*1*_, *β*_*2*_, *β*_*3*_, were set to very small. In this case, the lag effects could not be generated via Monte Carlo simulation at all.

**Figure 7:**
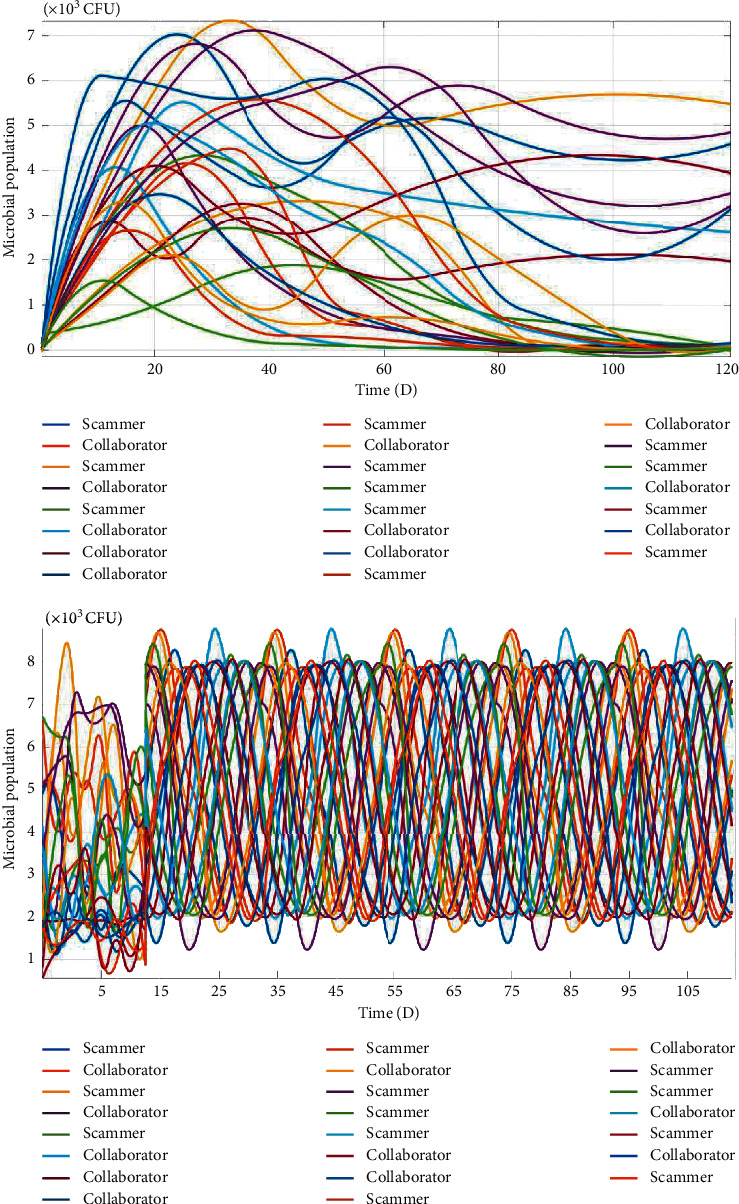
Dynamic response characteristics of oral microbial populations without (top) and with (bottom) delay effect community spatiotemporal succession process in oral cavity.

The time-domain response characteristics of oral microbial flora succession without and with lag effects could be obtained through accumulation of all individuals of the same species at different positions at the same time, similar to double integral in 2D cellular space (Figure 7).

From Figure 7 (top), all populations would grow exponentially at the beginning of microbial community succession, but their growth rates would have to slow down inevitably as niches are continuously filled up in oral cavity, and a turning point would appear sooner or later due to species differences in intrinsic growth rates, competitive capabilities, and carrying capacities. Some species would stop growing, whereas other species would keep increasing, and the latter would further exclude the former to make them extinct eventually (Hardin, 1960; Ives and Carpenter, 2007); eventually, only a few kinds of dominant species could coexist via transient responses, and their populations would asymptotically stabilize at a fixed level. For most oral microbial species, however, it would go extinct due to competitive exclusion and oral microbial diversity could not be formed and maintained at all.

**Figure 8:**
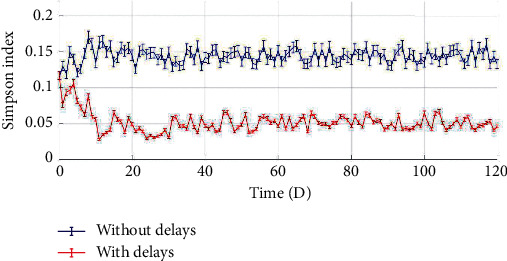
Simpson *α* index dynamics of oral microbial community without and with delay effects.

From Figure 7 (bottom), the digital simulation results illustrated that the most of oral microbial species could coexist, and their populations appeared periodical vibrations with shifted phases via unordered transient responses, forming an oral microbial climax community with higher richness and evenness (Figure 8). Because the microbial populations are asynchronously convergent fluctuations, as one species population is at a relatively high level, the other species populations might at relatively low levels due to phase differences, and these dynamic response characteristics could effectively reduce their interspecific competitions through minimization of interspecific competitive strengths which are mainly dependent on the product of microbial populations size, according to the classic *Lotka–Volterra* equations.

It is worth mentioning that the spatiotemporal succession patterns (Figures 5 and 7) of oral microbial flora were quite general and universal, this is because these emerging spatiotemporal patterns were insensitive to initial values of state variables and parameters in the CA model.

## 4. Discussion

Generally speaking, oral microbial delay responses are mainly caused by the oral immunity [16]. For example, the lysozyme and salivary cytokines such as IL-6, IL-17, IL-10, and TNF-*α* adversely influenced microbial cell division [21, 22]. From an ecological point of view, the oral immunity could be considered as an intermediate disturbance factor for the oral microbial community, which could effectively inhibit the overgrowth of dominant species and prevent nondominant species from going extinct. Hence, the intermediate disturbance could greatly increase evenness of oral microbial flora to enhance the microbial diversity. However, oral immunity did not kill oral microbial individuals directly but induced them to produce delay responses. Driven the decomposition product negative feedback regulation and delay responses, the microbial populations could present the asynchronously convergent fluctuations to effectively alleviate interspecific competition. In such circumstance, each microbial population would stay in a nonequilibrium state, and the microbial populations would start a new round of fluctuation to avoid going extinct due to competitive exclusion.

In the future research, oral microbial flora will need to be cultivated in the laboratory, and in-depth research studies will be carried out to elucidate the mechanism of microbial delayed responses from physiological, biochemical, and genetic levels in the emulated oral environment. Based on this study, specific medicines might be developed to enhance the oral immunity for promotion of the delayed responses of oral microorganisms [23].

## 5. Conclusion

Based on oral microbiology, microbial ecology, and experimental phenomena, a new hypothesis on formation and maintenance mechanism of oral microbial diversity was put forward and sufficiently confirmed by CA modeling and simulation in combination with experimental data, which demonstrated some oral microbial species such as *Haemophilus* and *Veillonella,* which could acclimate for oral environment with higher specific growth and substrate decomposition capability, while other species such as *Streptococcus*, *Rothia*, and *Prevotella* have no or weak adaptability with lower higher specific growth and substrate decomposition capability, which could form the decomposition product negative feedback regulation on microbial population size. In addition, the intracellular triglyceride accumulation of microbial species could produce reproductive and reaction delay responses in adversity, accompanying with interspecific competition and substrate decomposition delay responses.

Driven by decomposition product feedback regulation on microbial population sizes and four microbial delay responses, (1) from the view of time, oral microbial populations show asynchronously convergent fluctuations, significantly alleviating the interspecific competitions and inhibiting the emergence of dominant species. The oral microbial diversity could be formed and maintained by a strategy of species-for-quantity exchange; (2) from the view of space, the closely related microbial species would tend to aggregate in patches with different sizes, which also helped to further alleviate the interspecific competition strengths.

Hence, this study could not only lay the theoretical foundation for understanding of oral microbial diversity formation and maintenance but also offer effective guidance of prevention and treatment of oral cavity diseases.

## Figures and Tables

**Table 1:** Point estimation and 95% interval estimation of reproductive delay and reaction delay of the microbial population in oral cavity (unit: h).

Strains	Reproductive delay	Reaction delay
*Streptococcus*	2.85 ∈ [1.72, 3.92]	2.95 ∈ [1.88, 3.57]
*Prevotella*	2.45 ∈ [1.29, 3.98]	5.29 ∈ [4.50, 8.05]
*Haemophilus*	4.02 ∈ [2.18, 5.56]	8.53 ∈ [6.38, 10.07]
*Rothia*	2.85 ∈ [2.12, 3.92]	2.17 ∈ [1.88, 3.57]
*Veillonella*	3.28 ∈ [2.20, 4.18]	7.04 ∈ [4.50, 8.15]

**Table 2:** Parameters in the CA model of microbial community succession in oral cavity.

Name	Parameter	Range
Probability of entering lag phase	*α*_1_	[0.7, 1]
Probability of entering lag phase	*α*_2_	[0.3, 0.7]
Probability of entering lag phase	*α*_3_	[0, 0.3]
Probability of entering lag phase	*β*_1_	[0, 0.3]
Probability of entering lag phase	*β*_2_	[0.3, 0.7]
Probability of entering lag phase	*β*_3_	[0.7, 1]
Probability of move	*m*	[0, 0.2]
Probability of death exceeding lag phase	*p*	[0.8, 1]
Probability of natural death	*d*	[0.1, 0.3]
Lag phase time	*Lat*	[6, 10]
Maximum factor	*n*	[1, 3]
Initial kinds of microbial species	*N*	[5·10^2^, 10^3^]
Ration of *collaborator* richness to richness of initial microbial community	*N*_*p*_	[0.4, 0.6]
Initial population of each microbial species	*M*	[10^4^, 10^5^]
Ration of *collaborator* cells to the total number of microbial cells	*M*_*p*_	[0.3, 0.7]

## Data Availability

The data used to support the findings of this study are available from the corresponding author on request.
